# Reading about minds: The social-cognitive potential of narratives

**DOI:** 10.3758/s13423-022-02079-z

**Published:** 2022-03-22

**Authors:** Lynn S. Eekhof, Kobie van Krieken, Roel M. Willems

**Affiliations:** 1 Centre for Language Studies, Radboud University, Nijmegen, the Netherlands; 2 Donders Institute for Brain, Cognition and Behaviour, Radboud University, Nijmegen, the Netherlands; 3 Max Planck Institute for Psycholinguistics, Nijmegen, the Netherlands

**Keywords:** Narrative, Reading, Social cognition, Empathy

## Abstract

It is often argued that narratives improve social cognition, either by appealing to social-cognitive abilities as we engage with the story world and its characters, or by conveying social knowledge. Empirical studies have found support for both a correlational and a causal link between exposure to (literary, fictional) narratives and social cognition. However, a series of failed replications has cast doubt on the robustness of these claims. Here, we review the existing empirical literature and identify open questions and challenges. An important conclusion of the review is that previous research has given too little consideration to the diversity of narratives, readers, and social-cognitive processes involved in the social-cognitive potential of narratives. We therefore establish a research agenda, proposing that future research should focus on (1) the specific text characteristics that drive the social-cognitive potential of narratives, (2) the individual differences between readers with respect to their sensitivity to this potential, and (3) the various aspects of social cognition that are potentially affected by reading narratives. Our recommendations can guide the design of future studies that will help us understand how, for whom, and in what respect exposure to narratives can advantage social cognition.

One of the things that make us unique as human beings is our urge to communicate with each other by means of narratives (Boyd, 2009). From ancient myths to bedtime stories, and from narrative commercials to works of literary fiction: narratives are omnipresent throughout the lifetime. Unsurprisingly, then, reflections on the function of these narratives have likewise occupied countless readers, writers, and scholars. The social and emotional potential of narratives has led some to argue that exposure to narratives can strengthen our abilities to understand others (e.g., Mar & Oatley, 2008; Nussbaum, 1995, 2010). This suggests that the role of narratives transcends simple entertainment, potentially affecting personal lives as well as societies.

Empirical research seems to support the thesis that exposure to narratives improves our ability to understand others. Correlational studies, for instance, show that frequent exposure to literary fiction (in adults) or story books (in children) is associated with superior social-cognitive abilities (e.g., Adrian et al., 2005; Mar et al., 2006; see also Mumper & Gerrig, 2017). Furthermore, in an attempt to establish the causal direction of this association, several intervention studies as well as experiments found evidence for a direct, positive effect of a *single exposure* to literary narratives on social cognition (e.g., Black & Barnes, 2015b; Kidd et al., 2016; Kidd & Castano, 2013, 2018; Montgomery & Maunders, 2015; Pino & Mazza, 2016; van Kuijk et al., 2018). The general finding from these latter studies is that performance on social-cognitive measures increases immediately after reading a literary, fictional narrative, but not after reading a piece of popular fiction, nonfiction (e.g., an expository text), or nothing at all. However, three recent replication attempts did not find any significant direct effect of exposure to literary fiction compared with any of the other categories, and these failed replications have cast doubt on the social-cognitive benefits of narratives (Camerer et al., 2018; Panero et al., 2016; Samur et al., 2018).

We believe that the current state of mixed findings calls for reflection first, rather than more data. After discussing the conceptual background of what we will call *the social-cognitive potential of narratives*, we will give an overview of the existing empirical literature on both long-term associations between reading habits and social-cognitive abilities, and experimental research on the direct benefits of exposure to narratives, focusing mostly on research in neurotypical populations. Although much work has been done in the past years, several open questions and challenges remain unsolved. By identifying and critically discussing these, we aim to clear the ground for studies that will provide novel and nuanced insights in the relationship between narrative reading and social-cognitive abilities.

## Theoretical background

The idea that exposure to narratives can strengthen our social-cognitive abilities is articulated by psychologists (e.g., Mar & Oatley, 2008; Oatley, 1999), philosophers (e.g., Nussbaum, 1995, 2010), as well as literary scholars (e.g., Hakemulder, 2000; Zunshine, 2003, 2006), and can be traced back to work as early as Aristoteles’ *Poetics* (approx. 335 BC). Before we explain why these scholars have argued that narratives can strengthen our social abilities, we first need to clarify the concepts of narrative and social cognition.

### Narrative

Defining what constitutes a narrative, and what does not, has been the center of many debates among narratology scholars (see e.g., Rudrum, 2005; Ryan, 2007). In its most basic form, a narrative is often defined as a depiction of a sequence of related events in time (e.g., Abbott, 2008; Abrams & Harpham, 2009; Toolan, 2001). More elaborate definitions additionally stress the subjective nature of narratives (e.g., Bal, 2009). That is, narratives do not simply represent a sequence of external events but also imply the presence of a “subject of consciousness” who experiences the story events (Pander Maat & Sanders, 2002; Sanders & Redeker, 1993; Sanders, 2017). Readers are granted access to the inner world of these subjects through the use of viewpoint or perspective techniques—that is, the various linguistic means (e.g., verbs of cognition, descriptions of emotions) by which a writer or narrator “grant[s] us access to the internal and subjective viewpoints of characters within a narrative” (Eekhof et al., 2020, p. 2). On such accounts, “narrative is about human experience” (Ryan, 2007, p. 24) and “deals with the vicissitudes of human intentions” (Bruner, 1986, p. 16).

Although the term *narrative* is often used interchangeably with *fiction* and *literature*, strictly speaking fictionality and literariness are two dimensions that narratives can vary on independently. For example, narratives can be either fictional, as in the case of fairytales or romance novels, or nonfictional, as in the case of narratives based on true events, such as biographies (Abrams & Harpham, 2009). Similarly, both fictional and nonfictional narratives can be deemed literary (e.g., award-winning literary novels or biographies) or nonliterary (e.g., fan fiction written by teenagers, travel blog stories).

The distinction between the latter two, however, is hard to qualify objectively. From an extrinsic point of view, literary works may be contrasted with a category such as popular fiction based on social constructs of literariness, such as expert ratings, literary prizes (Gavaler & Johnson, 2017; Kidd & Castano, 2013; Koopman & Hakemulder, 2015), or author prestige and social consensus (Koolen et al., 2020). Scholars of Russian formalism, on the other hand, have attempted to formulate text-intrinsic characteristics of literary texts, arguing that the literary quality of a text can be found in its use of unconventional and defamiliarizing language, also called foregrounding (Abrams & Harpham, 2009; Gavaler & Johnson, 2017; Koopman & Hakemulder, 2015; Shklovsky, 1917/2004). The use of foregrounding devices, such as figures of speech, has been argued to uniquely draw attention to the formal aspects of the text, rather than the communicative message (Abrams & Harpham, 2009).

For the sake of transparency, we will use the word narrative to refer to any text that represents a sequence of events as experienced by a subject (see definitions above), regardless of the fictional and literary quality of these texts, while the term nonnarrative text refers to a text that does not represent a sequence of events as experienced by a subject but is expository in nature instead (e.g., an essay or encyclopedia article).

### Social cognition

Like narratives, social cognition also concerns the human experience and refers to the cognitive abilities people use “to make sense of other people and themselves” (Fiske & Taylor, 2013, p. 1). Two important social-cognitive processes that have been studied extensively, both on their own and in relation to narratives, are empathy and theory of mind. Empathy is a complex and multidimensional construct (Burke et al., 2016) that is often used to describe a broad array of processes, ranging from emotional contagion to compassion (Batson, 2009). By implication, the exact definition of empathy is a topic of debate. For example, de Vignemont and Singer (2006) define empathy as a vicarious experience by which we come to share the feelings of someone else, while still being aware that the source of these feelings lies outside ourselves. Embodied accounts have defined empathy as “a kind of direct, noninferential, (quasi-)perceptual awareness,” but not necessarily sharing, “of other people’s emotions, sensations, and other psychological states” (Zahavi & Overgaard, 2012, p. 16).

Unlike empathy, theory of mind, which is also referred to as mindreading, mentalizing, or folk psychology, denotes a more cognitively effortful process that allows us to understand the mental states of others and predict their behavior accordingly (de Vignemont & Singer, 2006; Frith & Frith, 2006). This understanding has been argued to come about either through the use of a set of rules that constitute a folk-psychological theory (theory theory; e.g., Gopnik & Meltzoff, 1997) or by putting ourselves in the others’ shoes through a process of simulation (simulation theory; e.g., Goldman, 1992; Gordon, 1986). Compared with empathy, theory of mind often seems to be reserved for the realm of cognitive mental states (i.e., beliefs and desires; e.g., Apperly, 2010), rather than the affective dimension. Yet the terminology used is far from transparent, as other researchers use the term cognitive empathy to refer to both cognitive and affective theory of mind (i.e., the active and effortful attempts to understand the cognitive and affective mental states of others). In this context, it is distinguished from emotional empathy, i.e., the more or less spontaneous sharing of emotions (Dvash & Shamay-Tsoory, 2014). All in all, empathy and theory of mind are hard to define concepts. Throughout this article we will therefore refer to “social cognition” as a general, umbrella construct, unless the studies we discuss have made claims about specific social-cognitive abilities.

### The social-cognitive potential of narratives

Having discussed these definitions, a clear connection between narrative comprehension and social cognition arises: both are centered around accessing and understanding the minds of others, be it narrative protagonists or people we encounter in the real world. This connection is the basis of various theories that suggest that exposure to narratives could foster social-cognitive abilities. The rationale for these theories mostly rests on either the activation of social-cognitive processes during narrative reading (process-based theories; Mar, 2018), or the transfer of knowledge through the narrative content (content-based theories; Mar, 2018). We will now discuss both positions in turn.

Process-based accounts are based on the idea that the brain uses the same cognitive systems to understand the minds of real and fictional others (in the case of emotions, this is sometimes called the “Panksepp-Jakobson hypothesis”; Jacobs, 2015). On such accounts, reading narratives is argued to draw on our real-life social-cognitive abilities (for neural support for this claim, see Mar, 2011). For example, Zunshine (2003, 2006) posits that we employ our mindreading or theory of mind skills to infer the mental states of narrative characters based on the descriptions of their behavior (see also van Duijn, 2018). In addition, Oatley (1999) describes narratives as a series of cues to run a mental simulation of the plot and, importantly, its corresponding emotions.

Interestingly, some scholars have also reasoned the other way around, arguing that social cognition involves the use of narrative processes. For example, Apperly (2010) describes mindreading as a process of creating situation models similar to those readers construct during narrative comprehension (e.g., Zwaan et al., 1995). Similarly, Ryan (2007) writes that “narrative involves the reconstruction of minds. But we perform this operation as a normal part of social life. Does it mean that we engage in private storytelling whenever we interact with human beings?” (pp. 27–28)

In line with these ideas, researchers have theorized that social-cognitive processes can be strengthened through their repeated use during reading (e.g., Mar, 2018). Mar and Oatley (2008), for example, argue that narrative “simulations of social experience” activate and train our empathic abilities by inviting us to try to understand and embody the emotions and beliefs of others in a process of what Koopman and Hakemulder (2015) have later termed “empathic imagination.” The recent SPaCEN (Social Processes and Content Entrained by Narrative) framework (Mar, 2018) aptly sums up the rationale behind the process-based theories by arguing that narratives can enhance social cognition if they “represent the social world” (p. 459) and activate social processes that can be developed through repeated practice. For example, frequently reading novels centered around romantic relationships might elicit our theory of mind as we try to understand what the underlying beliefs, intentions, and feelings of the characters are. Over time, his cognitive exercise might translate into improved cognitive theory of mind abilities.

The other, content-based strand of accounts have proposed that narratives (also) contribute to social cognition by conveying social *knowledge* (Mar, 2018; Mar & Oatley, 2008). For example, through narratives we might find ourselves in unique situations that we would normally never be able to experience, opening the door to a whole range of new (social) experiences and accompanying knowledge (Hakemulder, 2000; see also Montgomery & Maunders, 2015). In terms of the SPaCEN framework (Mar, 2018), this means that narratives can foster social cognition if they contain useful, learnable, and applicable knowledge about the social world. For example, reading a narrative about a break-up might provide us with knowledge about the dynamics of human relationships that can help us understand the relationships in our personal lives.

It is very probable that these two routes, elicitation of social processes and transmission of social knowledge, work alongside each other in practice. However, one could argue that the elicitation of social processes is what uniquely sets narratives apart from nonnarrative or expository texts. After all, expository texts can also contain social information (e.g., a handbook on couples counseling).

As Mar (2018) notes, most theoretical accounts of the social-cognitive potential of narratives have not been specific about the underlying time scale of the supposed relationships. That is, most theories do not elaborate on the amount of exposure to narratives needed to affect social cognition, nor specify how long effects last. The SPaCEN model (Mar, 2018), however, explicitly presupposes that frequent and prolonged exposure to narratives is needed to produce lasting impact, much like training a muscle involves repeated use of that muscle. In addition, most theories do not specify in what stages of readers’ lives or development beneficial effects of narratives on social cognition are to be expected (but see Mar, 2018, which will be discussed later on). This will be relevant when reviewing the empirical evidence in favor of these effects.

Moving beyond the idea that narratives in general improve social cognition, some scholars have made claims about literary and/or fictional narratives in particular. Theoretical accounts stressing the importance of literariness propose that the use of foregrounding in literary narratives specifically (i.e., the deviating use of language as a stylistic device in literature) elicits deeper forms of processing, reflection, and emotional response (Bálint et al., 2016; Sanford & Emmott, 2012). In line with this idea, Djikic and Oatley (2014) propose that literary features of a text can temporarily destabilize the personality and emotional system of the reader, which then allows for changes brought about by the narrative content.

Furthermore, scholars have argued that the complexity of literary texts requires extra (social-)cognitive efforts during processing and might thus lead to enhanced social-cognitive abilities. For example, literary fiction has been argued to be more layered, ambiguous, and less predictable, forcing the reader to engage in more (social) inferencing (Kidd & Castano, 2013). In addition, Zunshine (2011) argues that aspects of literary style, such as metaphors and other figures of speech, lead to a certain kind of social-cognitive complexity—for example, by making the reader aware of the subtle intentions and expectations of the narrator (see also Gibbs & Colston, 2019). Taken together, these accounts propose that literary narratives contain more social-cognitive complexity and as such provide a greater “work-out” for readers’ social cognition, leading to greater benefits compared with nonliterary narratives.

Yet other theorists have emphasized the role of fictionality, arguing that fictional narratives create a beneficial distance to the real world (Hakemulder, 2000; Keen, 2007; Oatley, 1999). This “protective fictionality”, as Keen (2007, p. xiii) calls it, means that readers can let their guard down and empathize with the narrative experiences without facing real-life consequences (Hakemulder, 2000). As a result, fictional narratives would allow readers to engage in “safer” and thus more perspective-taking than nonfictional narratives, potentially leading to bigger effects on social cognition.

In summary, (frequent) exposure to narratives has been hypothesized to promote social cognition through the activation and subsequent strengthening of social-cognitive processes and through the transfer of socially relevant information. Furthermore, literariness and fictionality have been mentioned as additional driving forces behind this effect. As we will see in the next section, in more recent years, empirical researchers have begun to test these hypotheses. In what follows, we will discuss the existing empirical literature on the relationship between narratives and social cognition by looking both at the associations between reading habits and social-cognitive abilities as established in correlational and longitudinal studies, and the causal effects of exposure to narratives, as studied in experiments and interventions.

## Empirical evidence

### Correlational and longitudinal studies

One line of research on the relationship between narrative reading and social-cognitive abilities has looked at associations between reading habits and various measures of social cognition. Researchers found positive relationships in age groups as young as preschoolers in both cross-sectional studies (e.g., Adrian et al., 2005; Aram & Aviram, 2009; Mar et al., 2010) and longitudinal studies (e.g., Rose et al., 2018). In these age groups, exposure to narratives is usually measured either explicitly, by asking caregivers how often they read books to their child, or more implicitly, with the use of recognition tests. In such tests, participants, in this case caregivers, are asked to indicate which author names (Author Recognition Test; ART; Stanovich & West, 1989), book titles, or phrases they know from a list that is made up of both existing names, titles, and phrases and foils. Scores on such tests are argued to reflect exposure to (certain types of) print. For example, Aram and Aviram (2009) measured mothers’ ability to recognize key phrases and authors of children’s books, supposedly reflecting the frequency with which they read these books to their children. They then found that scores on this measure were positively related to their children’s empathy level, as assessed by kindergarten teachers, even after controlling for mothers’ education level. Importantly, Mar et al. (2010) found that this relationship could not be explained by parents’ literacy in general, since only parents’ ability to recognize children’s book titles and authors, but not adult book authors, was related to theory of mind performance in 4- to 6-year-olds, even after controlling for age, gender, language abilities, and parental income.

Although these studies seem to suggest that exposure to narratives benefits social-cognitive development in children, the question remains whether the found relationships are solely due to narrative exposure or are rather also the result of the accompanying social interaction between child and caregiver that is often centered around the mental states of narrative characters (Mar et al., 2010; see also Ratner & Olver, 1998). For example, Adrian et al. (2005) found that not only the frequency of joint book reading, but also the frequency and variety of mothers’ mental state talk during reading was related to performance on false belief tasks. Hence, as young children’s exposure to narratives is usually embedded in a highly social context, it is difficult to disentangle the contribution of the narratives per se from the contribution of the surrounding social interaction.

Studies on children who can read by themselves might thus be better suited to study the relationship between social cognition and narrative exposure in a more restricted sense. However, social-cognitive development after early childhood has received relatively little attention (Kilford et al., 2016; but see Pavias et al., 2016). A recent study that did look at a large group of children from a wide range of age groups (8 to 16 years old) found a significant relationship between the frequency of exposure to fictional narratives, as measured with self-report questionnaires, and self-reported perspective-taking tendencies, while controlling for age and gender (De Mulder et al., 2021). However, no relationship was found with performance-based measures of emotion recognition (i.e., ability to assign the correct emotion label to a picture). Moreover, in a study with German adolescents, Lenhart et al. (2020) failed to find a relationship between fiction exposure, as measured with an author and title recognition test, and self-reported social-cognitive abilities when not only controlling for age and gender, but also for IQ and openness to experiences. De Mulder et al. (2021) suggest that a possible explanation for the lack of a clear relationship between fiction reading and social cognition in school-age children and adolescents is the fact that in these phases of life most reading takes place in educational contexts where exposure to fiction is compulsory. Interestingly, this hypothesis seems to be backed up by a longitudinal study by Mak and Fancourt (2020) who found that reading for pleasure at age seven, i.e., reading that is done outside of a school context, *was* associated with prosocial behavior at age 11, as measured with a parental questionnaire, even after controlling for a range of variables.

Finally, a number of studies have looked at the association between adults’ reading habits and their social-cognitive abilities. For example, Mar et al. (2006) found that exposure to fiction, as measured by the number of correctly identified names of fiction writers, was positively associated with scores on the Reading the Mind in the Eyes Task (RMET; Baron-Cohen et al., 2001), a word–picture matching task that measures the ability to ascribe affective mental states to pictures of eyes, even after controlling for age, English fluency, and intelligence. These results provide support for a positive relationship between exposure to fiction and emotion recognition abilities. Moreover, exposure to nonfiction was negatively associated with performance on this task and another task of interpersonal sensitivity, suggesting that exposure to nonfiction does not have a neutral but rather a potentially detrimental effect on social-cognitive abilities compared with exposure to fiction.

The long-term association between exposure to fiction and social-cognitive skills in adults has since been observed in multiple other studies, using a variety of measures (e.g., Black & Barnes, 2015b; Djikic et al., 2013; Fong et al., 2013; Mar et al., 2009; Schwering et al., 2021; for an overview, see Mumper & Gerrig, 2017). Moreover, in an fMRI study Tamir et al. (2016) found that the positive relationship between fiction exposure (ART) and performance on mindreading tasks was mediated by the degree to which the brain regions related to theory of mind were activated when participants read social narratives, providing support for the idea that social cognition develops through repeated activation of social-cognitive processes elicited by narratives.

In sum, evidence from correlational and longitudinal studies suggests that exposure to narratives is positively related to social-cognitive abilities in preschoolers and adults. The evidence for school-age children and adolescents is more mixed but is indicative of an association between noncompulsory reading for pleasure and social cognition. Nevertheless, these findings do not necessarily provide direct evidence for a true causal effect of narrative reading on social-cognitive abilities: it might well be that the positive association between exposure to fiction and social-cognitive skills reflects the tendency of socially competent people to turn to fiction reading more often, for example because they enjoy reading about the inner worlds of others in stories. Experiments and intervention studies were developed to further establish the causal direction of the relationship between reading narratives and social cognition and find additional support for the social-cognitive hypothesis of narrative reading.

### Experiments and intervention studies

The rationale behind most experimental studies assessing the causal effects of reading narratives is that if reading narratives leads to improved social cognition, then social-cognitive performance should be enhanced after exposure to narratives, but not after exposure to nonnarrative texts or no exposure to any text. One line of research based on this approach has used interventions to study the social-cognitive potential of narratives. In these studies, participants in the intervention group are repeatedly exposed to narratives over an extended period of time (e.g., a week up to several months). Social-cognitive abilities are measured both before and after the intervention, and improvements in abilities are compared between the intervention group and a control group.

Intervention studies have thus far mostly been used to study the social-cognitive potential of narratives in young populations, possibly because interventions are relatively easy to implement in an educational setting. For example, in an intervention study in German after-school childcare centers, 7- to 9-year-olds’ emotional vocabulary and their ability to identify, label and understand both visible and concealed feelings improved after eight 90-minute sessions of joint reading (Kumschick et al., 2014). In a review article, Montgomery and Maunders (2015) discuss eight other studies that report positive effects of narrative interventions, also called bibliotherapy, on various measures of social cognition and prosocial behavior in 5- to 15-year-old children. The downside of these intervention studies, however, is that exposure to narratives is usually accompanied by various activities such as discussion groups or creative exercises, making it difficult to assess what the actual contribution of the narrative exposure is.

Other researchers have used experiments to target the specific effect of exposure to (certain types of) narratives on social cognition. In these studies, the social-cognitive abilities of a group of participants who have been exposed to *one* particular kind of narrative are compared with the social-cognitive abilities of other groups that have been exposed to other types of texts (e.g., an expository text) or nothing at all. Using this approach, Djikic et al. (2013) found that participants who scored low on the personality trait “openness” experienced an increase in self-reported cognitive empathy (as measured with the self-report Perspective Taking scale of the Interpersonal Reactivity Index, IRI; Davis, 1983) after reading a literary story, but not after reading an expository text that was matched in terms of content, complexity, and length. The authors suggest that individuals who are generally not as open to new experiences benefit especially from the exposure to others’ perspectives that literary narratives offer, increasing their self-reported empathic abilities (see Djikic et al., 2009b).

As self-reported changes do not necessarily translate into actual abilities, a study by Kidd and Castano (2013) provided more evidence in favor of a direct effect of narrative reading on social-cognitive skills. In their experiments, the Reading the Mind in the Eyes Test (Baron-Cohen et al., 2001, see above) and the Yoni Task (Shamay-Tsoory & Aharon-Peretz, 2007) were used to measure participants’ social-cognitive abilities. The Yoni Task is a measure of cognitive and affective theory of mind that uses cartoons to assess the ability to infer the intentions and emotions of a character named Yoni based on verbal and eye-gaze cues. In a series of five experiments in which participants were assigned to read either an excerpt of literary fiction, popular fiction, nonfiction, or nothing, it was found that those who read literary fiction outperformed those who read popular fiction, nonfiction, or nothing on the Reading the Mind in the Eyes Test. Moreover, participants in the literary fiction condition outperformed those in the popular fiction condition on the Yoni Task. The authors thus concluded that engagement with narratives, in particular literary fictional narratives, enhances theory of mind.

Several studies have since attempted to replicate the immediate effect of a single exposure to literary fiction, with varying success. Some studies were able to replicate the positive effect of literary fiction on social-cognitive abilities as compared with the effect of popular fiction (Kidd & Castano, 2019; van Kuijk et al., 2018). In addition, exposure to literary fiction has also been found to have a positive effect when compared with science fiction, a genre closely related to popular fiction: students assigned to read a work of literary fiction outperformed a group of students who were assigned to read a work of science fiction on two theory of mind tasks after finishing the book (Pino & Mazza, 2016).

Moreover, the finding that reading a piece of literary fiction has a positive effect when compared with nonfiction has also been backed up by additional studies (Bal & Veltkamp, 2013; Black & Barnes, 2015a, 2015b; Pino & Mazza, 2016). For example, using a within-subjects design, Black and Barnes (2015a, 2015b) found that reading literary fiction significantly improved scores on the RMET compared with the effect of reading nonfiction. Moreover, performance on an intuitive physics understanding test was not affected by reading condition, suggesting that the positive effect of literary fiction cannot be explained as a general improvement of (nonsocial and social) cognitive abilities as a result of the complexity of literary texts. Thus, the authors conclude that there seems to be a unique, direct link between one-time exposure to (literary) narratives and *social* cognition, rather than cognition in general.

However, other studies, including some direct replications of Kidd and Castano’s (2013) experiments, have not found evidence for a direct positive effect of reading a piece of literary fiction as opposed to either popular fiction or nonfiction (Camerer et al., 2018; De Mulder et al., 2017; Panero et al., 2016; Samur et al., 2018; see also Djikic et al., 2012), causing many to cast doubts on the original claims. Nevertheless, a recent meta-analysis (Dodell-Feder & Tamir, 2018) that also included two of the recent failed replications (i.e., Panero et al., 2016; Samur et al., 2018), found that reading a piece of literary fiction does in fact have a small positive effect (*g* = .15–.16) on social-cognitive abilities (both when looking at all effect sizes and when looking exclusively at effect sizes obtained with the RMET) when compared with reading nonfiction or nothing.

Nonetheless, the single-exposure approach has received additional criticism recently, as the rationale behind studies using the experimental design described above seems to contradict the tacit assumption of the theoretical models that *repeated* exposure to narratives is needed to improve social-cognitive abilities. In his SPaCEN framework, Mar (2018) argues that the rationale of the single-exposure studies is too simple. That is, assuming that in a sample of healthy adults with at least some previous reading experience, a single exposure to a brief narrative would lastingly improve something as substantial as social cognition is naïve. Instead, the results from single-exposure experiments should perhaps be interpreted as narratives temporarily putting readers in the “mood” for mind reading or making readers more aware of the inner worlds of others (see also Manierka et al., 2021).

An additional problem that experiments face is that they almost exclusively make use of the RMET to measure social-cognitive abilities. Not only has the RMET been criticized for its poor internal consistency and homogeneity (Olderbak et al., 2015), a recent study also showed that performance on the RMET correlates highly with measures of verbal ability (Peterson & Miller, 2012). This is highly problematic for research on the relationship between narrative exposure and social cognition, because this means that any found effects might in fact reflect a positive effect of reading on verbal abilities (e.g., Mol & Bus, 2011), rather than social-cognitive abilities. Although this issue might be partially solved by controlling for language abilities, as some studies have done, results from experiments solely relying on the RMET should be interpreted with caution.

All in all, then, the best evidence in favor of a causal effect of reading narratives on social cognition comes from the intervention studies (Kumschick et al., 2014; Montgomery & Maunders, 2015) and a handful of experiments that have not solely relied on the RMET to measure social-cognitive abilities (i.e., Bal & Veltkamp, 2013; Djikic et al., 2013; Kidd & Castano, 2013; Pino & Mazza, 2016). However, even studies that have employed other measures than the RMET have not always replicated the positive effect of a single case of exposure of narratives on social cognition (e.g., De Mulder et al., 2017; see also Dodell-Feder & Tamir, 2018). Thus, experimental evidence for the social-cognitive potential of narratives is mixed at best and the question rises how these mixed findings should be interpreted.

We propose that part of the explanation for these conflicting outcomes might lie in the fact that previous studies have often collapsed various types of texts, readers, and social-cognitive processes, tacitly assuming that any (literary) narrative will affect all readers in the same, positive way. To overcome this generalized approach, there is a need of experiments that even more specifically isolate “narrative features that promote a positive impact on social cognition” (Mumper & Gerrig, 2017, p. 117). Moreover, more attention has to be paid to individual differences between readers, in an attempt to clarify what readers can benefit from the proposed positive impact and which specific aspects of social cognition are in fact impacted. In other words, rather than working from the idea that narratives either do or do not impact social cognition, we propose to work from the idea that narratives can impact social cognition in certain circumstances and focus on mapping out these circumstances.

We argue that in order to move forward, reflection is needed on the three central aspects of the social-cognitive potential of narratives: the text, the reader, and the social-cognitive processes. In the next section we therefore identify open questions and challenges related to these three aspects that can lead these further inquiries and help move the field forward. Ultimately, these reflections can lead to carefully constructed experiments that can help elucidate how, for whom, and when the social-cognitive potential of narratives emerges.

## Open questions and challenges

### What text characteristics drive the social-cognitive potential of narratives?

Most research designs that have been used thus far do not provide much insight in the specific textual characteristics that drive the positive effects of reading narratives. Studies have mostly focused on global text dimensions such as literariness and fictionality and have often resorted to making comparisons that conflate various textual dimensions, making it hard to draw sound conclusions about the driving factors behind any found differences. In this section, we will discuss these challenges in more detail, and provide avenues for future research on the textual characteristics that drive the positive effects of narrative reading.

Following the theoretical accounts that put a special emphasis on the general concepts of literariness and fictionality as the driving forces behind the social-cognitive potential of narratives (e.g., Keen, 2007; Zunshine, 2011), most empirical studies have aimed to investigate the difference between literary fiction, popular fiction, and nonfiction. As described above, some studies have found evidence for a beneficial effect of literariness by comparing the effect of reading a piece of literary fiction to the effect of reading a piece of popular fiction (Kidd & Castano, 2013, 2018; Pino & Mazza, 2016; van Kuijk et al., 2018). However, others have not been able to reproduce this finding (Camerer et al., 2018; Panero et al., 2016; Samur et al., 2018) and this approach has since been criticized (Gavaler & Johnson, 2017; Koopman & Hakemulder, 2015; Panero et al., 2016). One of the objections is that the texts in the original Kidd and Castano (2013) experiments were chosen based on extrinsic criteria, such as prizes and ranking (for an elaborate critique, see Gavaler & Johnson, 2017), and the various texts used in the different conditions were poorly matched on, for example, content. Hence, it is hard to disentangle exactly which intrinsic characteristics of the textual stimuli were responsible for the difference found between literary and popular fiction narratives (Gavaler & Johnson, 2017).

Other studies have attempted to demonstrate the specific effect of literariness and/or fictionality on social-cognitive abilities by comparing the effect of literary fiction to the effect of nonfiction (i.e., expository texts; Bal & Veltkamp, 2013; Black & Barnes, 2015a, 2015b; De Mulder et al., 2017; Kidd & Castano, 2013; Pino & Mazza, 2016). This comparison is problematic, however, as it collapses the effects of literariness, fictionality, and narrativity by comparing a literary, fictional narrative (literary fiction) to a nonliterary, nonfictional expository text (nonfiction). The evidential value of these studies is thus limited when evaluating and studying the textual causes behind the found differences.

One possible solution for this issue lies in studies that have used text manipulations to study the effect of specific literary features on social-cognitive processes. For example, Koopman (2016) found that readers who read a narrative that was high in foregrounding (i.e., containing literary devices such as metaphors, alliterations, ellipses, etc.) reported more empathic understanding than those who read a manipulated version without foregrounding of the same narrative. However, in a qualitative study by Kuzmičová et al. (2017), readers’ elaborations were in fact found to be more empathic after reading a manipulated narrative without foregrounding rather than after reading the original narrative high in foregrounding. Another study examined literary gaps, instances in the narrative where readers are invited to use social inferencing and creativity to complete missing information (De Mulder et al., 2017). The authors hypothesized that a narrative with literary gaps would boost social-cognitive abilities more than a manipulated narrative in which these gaps were already filled in. However, no effect of the presence of literary gaps on measures of theory of mind was found. In sum then, empirical research on literariness has yielded little evidence for its effect on social cognition, nor has it convincingly provided specific text characteristics that might drive the social-cognitive potential of narratives.

To our knowledge, empirical studies thus far have not isolated the specific effect of fictionality. An fMRI study, however, does suggest that brain regions related to emotion are more active when readers think they are reading a fictional narrative compared with a nonfictional narrative (Altmann et al., 2014), providing some initial support for the idea of protective fictionality.

Koopman and Hakemulder (2015) have argued that rather than focusing on literariness or fictionality, a more fruitful approach might be to study characteristics related to the overarching concept of narrativity (see also Mar, 2018), because the positive effect of narrative reading, when found, seems to extend to narratives in general (e.g., including life narratives; see Koopman, 2015). That is not to say that literariness and fictionality do not play a role at all. However, regardless of their literariness or fictionality, narratives can be distinguished from nonnarrative or expository texts in terms of form, content, and the type of engagement they bring about. These characteristics might be worthwhile to study in more detail in future research.

There is already some evidence that formal narrative characteristics, such as the representation of the inner world of protagonists, might play a role. For example, Kidd et al. (2016) found that the beneficial effect of literary fiction compared with popular fiction was mediated by “the extent to which a text provides sophisticated interpretations of behavior in terms of mental states” (p. 51), as measured by Computerized Reflective Function, which automatically analyzes a text for the presence of linguistic items that signal high levels of reflection (e.g., “think,” “but”) as opposed to low levels of reflection (e.g., “me,” “can”). Furthermore, Johnson, Jasper, et al. (2013b) found that empathy for Arab Muslims was significantly higher after reading a full narrative that included dialogues and monologues than after reading a condensed form of the same narrative, which was a shorter summarized version of the plot. Other characteristics that might be of importance include viewpoint or perspective markers (see, e.g., Eekhof et al., 2021; van Krieken et al., 2017) or descriptions of mental states in general (see, e.g., Cupchik et al., 1998; Gavaler & Johnson, 2017; Habermas & Diel, 2010). An unresolved question, however, is to what degree the presence of mental state descriptions is most beneficial to social cognition, and to what degree their relative absence within an otherwise complete narrative is in fact more constructive, because they require readers to put their mindreading and inferencing abilities to work. An intervention study with 4-year-olds provided some evidence for the latter, showing that children who were exposed to stories without mental state descriptions outperformed a group of children who were exposed to the same stories enriched with mental state descriptions on various false-belief tasks (Peskin & Astington, 2004).

Although no content is unique to narratives per se, there are indications that certain content, when expressed in a narrative form, has a stronger effect on social cognition. Narratives with social content lead to more activation in brain areas related to theory of mind, compared with nonsocial narratives (Tamir et al., 2016). In addition, especially narratives that convey negative emotion seem to engage these areas (Altmann et al., 2012). This finding is further supported by a correlational study that found that exposure to romance, a genre known to focus on relationships and emotions, more so than exposure to other fictional genres, was related to better performance on the RMET, even while controlling for various variables including English fluency, trait openness and extraversion (Fong et al., 2013). Other content-related aspects that might play a role include the number of characters (Kuzmičová et al., 2017), the morality or likeability of characters (Habermas & Diel, 2010; Salgaro & Tourhout, 2018), or the similarity between the character and the reader (Komeda et al., 2013). More research is needed to further explore the role of story content and its interaction with the narrative form in the social-cognitive potential of narratives.

Crucially, the narrative form is also known to elicit processes of narrative engagement, such as absorption (Kuijpers et al., 2014) or transportation (Green et al., 2004): the pleasurable feeling of “being lost” in a story world (Nell, 1988), as well as narrative empathy (Keen, 2007), and mental imagery. Future research could therefore also investigate the role of functional aspects of narratives (i.e., related to the experience) as opposed to extensional aspects (i.e., related to form/content; Tay et al., 2018). For example, Calarco et al. (2017) argue that absorption and identification might facilitate the social-cognitive potential of narratives: the more readers are absorbed in the narrative and align themselves with the characters, the more social processes might be activated and thus trained.

Differences in the extent to which narrative engagement is evoked during reading have already been found to modulate the effect of (literary, fictional) narratives on empathy and prosocial behavior (e.g., Bal & Veltkamp, 2013; Johnson, 2012, 2013; Johnson, Cushman, et al., 2013a; Johnson, Jasper, et al., 2013b; Stansfield & Bunce, 2014; Walkington et al., 2019). However, as Tay et al. (2018) point out in their model on the role of the arts and humanities in human flourishing: it remains to be seen whether these forms of engagement are mediators (i.e., text-dependent) or moderators (i.e., reader-dependent). In other words, it is not clear yet whether certain narratives might bring about a form of narrative engagement that consequently positively impacts social cognition, or whether readers with a higher disposition for this type of engagement (e.g., high transportability) benefit more from exposure to narratives.

The studies discussed above give an impression of the narrative characteristics that may play a role in advancing social-cognitive abilities through narrative exposure. As became apparent from the discussions, the main challenge lies in designing research designs that can help move the study of the driving factors behind the social-cognitive potential of narratives beyond the broad concepts of literariness and fictionality. Crucially, this might call for new experimental approaches, such as textual manipulations, within-subject designs, or methodologies such as eye-tracking or other methods that allow for the measurement of online effects of word-level characteristics. Finally, as narrativity can be distinguished from literariness and fictionality, a broader range of narratives should be included in future research. For example, nonfictional narratives, both of literary quality (e.g., biographies, memoirs, literary journalism; van Krieken, 2019) and nonliterary quality (e.g., personal narratives) could be studied to see how social-cognitive abilities are impacted by engaging with narrative accounts of real-life events.

### What types of readers are susceptible to these effects?

The effect of exposure to narratives likely does not only vary as a function of textual characteristics, but also depends on characteristics of the reader and the interaction between the text and the reader (see also Gerrig & Mumper, 2017). Some scholars have even argued that the match between the reader and the text might be more important than the text itself (Tay et al., 2018). Nevertheless, previous research has mostly only controlled for individual differences in trait empathy and print exposure between adult readers (e.g., Kidd & Castano, 2013), or differences in demographic variables such as age and parental income between children (e.g., Mar et al., 2010). Relatively few studies have looked at these and other individual differences as factors of interest and this might partially explain the mixed findings observed thus far: by lumping together a heterogeneous sample of participants into a single “idealized reader,” we might miss the possibility that readers with different characteristics react differently to the same text. In this section, we will discuss opportunities for future research related to individual differences between readers and their susceptibility to the social-cognitive potential of narratives.

Several studies provide evidence for the role of individual differences in the relationship between narrative reading and social cognition. As described above, a beneficial effect of reading literary fiction over nonfiction was found for readers with low scores on the openness dimension of the Big Five Inventory, but not for readers high in openness (Djikic et al., 2013). In a similar study, readers with a highly avoidant attachment style were found to experience more emotion change after reading an excerpt of literary fiction than after reading a matched expository text, whereas the difference between the two texts was not significant for readers with a less avoidant attachment style (Djikic et al., 2009a). A study on the long-term associations between reading habits and social cognition also reported that, after controlling for multiple other individual differences, a positive association between exposure to narrative fiction and empathic concern was only found for high school students with a low tendency to become transported into narrative worlds (i.e., low transportability; Lenhart et al., 2020). Together, these findings seem to suggest that exposure to narratives is especially beneficial to readers who have a tendency to avoid emotional situations. That is, readers who normally have a hard time opening up to emotional experiences or might even resist such experiences, might feel safe to let their guard down when reading narrative representations of emotional situations and subsequently benefit more from doing so than those who already find themselves in emotional situations regularly in daily life.

In addition, age and social-cognitive development might play a role in how sensitive readers are to the benefits of narrative exposure. Mar (2018) argues that the degree to which readers’ social-cognitive abilities are receptive to change might vary with age, such that large effects of narrative exposure could be expected in children and adolescents (Kilford et al., 2016) whose social cognition is still in the midst of development. While adults on average might have less room for improvement, exposure to narratives might still affect those with relatively large opportunity for development, such as those who with an autism spectrum disorder (see Tsunemi et al., 2014). To further understand how social cognition might be fostered through narrative exposure across the life span, more research is thus needed to understand what aspects of social cognition are receptive to what degrees of improvement in various stages of development (see also Mar, 2018). Note that at least in the case of empathy, there is evidence that adults can still improve their empathic skills through various training interventions (e.g., role-play activities; Bas-Sarmiento et al., 2020; Teding van Berkhout & Malouff, 2016; Weisz et al., 2021).

Although, on the one hand, some room to grow might be needed for the social-cognitive potential of narratives to arise, some basic level of social-cognitive abilities might, on the other hand, already be needed to be able to understand and thus benefit from narratives. For example, Pavias et al. (2016) showed that the ability to recall socially relevant aspects of narratives increases with age, especially during adolescence, potentially mirroring developments in social cognition (see also Sebastian et al., 2012). Moreover, even within healthy adults social-cognitive abilities affect narrative processing (Eekhof et al., 2021). However, given that positive effects of reading have been found in children as young as three years (e.g., Rose et al., 2018), these minimally required abilities might be in place already at early stages of social-cognitive development.

Similarly, individual differences in verbal and reading abilities might play a role. Readers who have a hard time reading and understanding a narrative, might not be able to form rich simulations of the story world and character’s minds. Indeed, various studies have found that readers with higher print exposure scores find it easier to emotionally engage with story characters (Koopman, 2015, 2016; van Lissa et al., 2018). Thus, a certain level of reading abilities might need to be in place in readers, possibly depending on the complexity of the narrative as well, in order for the social-cognitive potential of narratives to arise.

Besides these trait-related individual differences, a study by Koopman (2015) suggests that personal experience with the topic of a narrative leads to more prosocial behavior and empathic understanding: participants who had personal experience with depression were more likely to donate money to charity and reported more understanding for depressed patients after reading, regardless of the genre of the text they had just read (see also Green, 2004). The author suggests that readers with personal experience with a topic might be more engaged by a story, potentially leading to more activation of social-cognitive processes. This idea is backed up by an fMRI study by Chow et al. (2015): not only did readers report more vivid imagery when they had personal experience with the situations described in a story, it was also found that connectivity within motor and visual regions increased with personal experience, suggesting that personal experience leads to richer or deeper forms of narrative engagement.

In a similar vein, some researchers have also suggested that there might be a role for personal preferences (e.g., De Mulder et al., 2021; Djikic et al., 2012; Panero et al., 2016), such that when readers are allowed to choose what narrative they want to read, more positive effects might be observed, again because narrative engagement seems to facilitate the effect of reading on social cognition (see previous section).

To conclude, future studies should focus on the characteristics that make readers more or less sensitive to the social-cognitive potential of narratives in general and in relation to specific types of narratives and textual characteristics. Including measures of individual differences in experiments might reveal interesting patterns of sensitivity in heterogeneous groups of readers that might otherwise have been overshadowed by the absence of significant main effects of narrative exposure. Besides emotional disposition, social-cognitive development, verbal abilities, personal experience and preference, additional relevant characteristics that have been found to play a role in other narrative processes include the need for affect (Maio & Esses, 2001) and the need for cognition (Cacioppo & Petty, 1982; see also Appel & Richter, 2010; Green et al., 2008; Kuijpers et al., 2019). Finally, the individual differences approach will not only advance our understanding of the precise workings of the social-cognitive potential of narratives but will also open up the possibility of reliably and strategically putting this potential into practice, for example in patient populations that need additional empathy training (Calarco et al., 2017).

### Which aspects of social cognition are influenced by narrative reading?

Following theoretical accounts on the social-cognitive potential of narratives, most empirical studies have focused on the relationship between narrative reading and the broad concepts of empathy and theory of mind. Future studies should aim for both a deeper and broader view on the aspects of social cognition that narratives might influence. In this section we will discuss the practical and theoretical challenges that come with this line of research.

One of the primary challenges that empirical studies of the social-cognitive potential of narratives have faced is to translate theoretical claims about the effects of narratives on social cognition into experiments that test how specific, quantifiable social-cognitive abilities are affected by exposure to narrative. This is difficult for two reasons. First of all, it is not always clear what a specific task measures, or, vice versa, how a certain ability can be measured in a valid way. For example, the Reading the Mind in the Eyes Task (RMET; Baron-Cohen et al., 2001) has been used to make claims about a broad variety of abilities (Stansfield & Bunce, 2014), ranging from emotion recognition (van Kuijk et al., 2018) to empathy (Djikic et al., 2013), cognitive empathy (Mar et al., 2006), and affective theory of mind (Kidd & Castano, 2013). As a result, it is hard to draw sound conclusions on the specific aspects of social cognition that are impacted by exposure to narratives.

Secondly, Turner and Felisberti (2017) have noted the lack of tasks that can reliably measure the subtle differences in mindreading abilities that can be expected among healthy adults. They argue that most tasks that are available suffer from ceiling effects, as they were originally designed to be used in clinical and developmental contexts, for example to distinguish those with autism spectrum disorders from healthy controls (see also Black, 2019). In general, then, an important avenue for future research is to develop tasks and measures that can support more specific claims about the relationship between narratives and particular social-cognitive abilities.

Another important avenue for future studies involves broadening the scope of social-cognitive abilities under investigation beyond empathy and theory of mind. As Mar (2018) has shown in his SPaCEN framework, the proposed mechanism behind the relationship between narrative reading and empathic and mindreading abilities can be applied to a range of aspects of social cognition, as long as these abilities depend on either trainable processes that are activated by narrative reading or knowledge that narratives can convey.

Empirical research on the effects of narrative exposure on other social-cognitive abilities is relatively scarce thus far but provides some promising leads. Exposure to narratives has been found to increase certain behaviors that might depend on social-cognitive abilities, such as prosocial behavior (Johnson, 2012; Koopman, 2015). For example, readers who reported feeling high levels of affective empathy for the main protagonist of a narrative during reading were twice as likely to help the experimenter pick up dropped pens than those who reported low levels of affective narrative empathy (Johnson, 2012). Reading a narrative can also reduce prejudice and stereotyping (Hakemulder, 2000; Johnson, 2012, 2013; Johnson et al., 2013a, b; Koopman, 2015; Vezzali et al., 2015; see also Fong et al., 2015, for long-term effects on sexual stereotyping). For instance, readers who were transported in a narrative describing the experiences of an Arab Muslim woman reported less stereotypical beliefs about Arab-Muslims afterwards and experienced more positive attitudes (Johnson, 2013). This effect was mediated by the degree to which participants experienced affective empathy towards the protagonist of the narrative.

In addition to empathy and theory of mind, which have been the primary focus of research thus far, prosocial behavior and stereotyping, which have started to gain more interest, future research could study the effect on other social-cognitive abilities related to understanding others, such as emotional contagion, emotion recognition, emotion regulation, social memory, social schemas, facial recognition, or even processes related to understanding the self (see also Mar, 2018). When we have a more detailed understanding of the various social-cognitive abilities that are positively (or negatively) affected by narrative reading, this will also clear the ground for clearly targeted interventions in populations suffering from specific social-cognitive deficits.

## Conclusion

Inspired by reflections on the function of narratives, recent years have seen a rise in studies looking at the relationship between narrative reading and social cognition. A review of the empirical literature on both the correlations between reading habits and social-cognitive abilities and the causal effects of narrative exposure on these abilities shows conflicting findings: although the long-term associations are rather stable, reading a single narrative sometimes does and sometimes does not lead to improved social-cognitive abilities compared with reading nonnarrative expository texts or nothing, and this approach has recently received criticism. Ultimately this means that the question “does narrative reading promote social-cognitive abilities?” cannot be answered unequivocally. However, another way of looking at these conflicting findings might be to think of narratives as having a social-cognitive potential that sometimes does and sometimes does not arise. In this paper, we have argued that future research should focus on mapping out the circumstances that allow this potential to come about by focusing on specific aspects of the reader, the text, and social cognition (see also Panero et al., 2016).

**Fig. 1:**
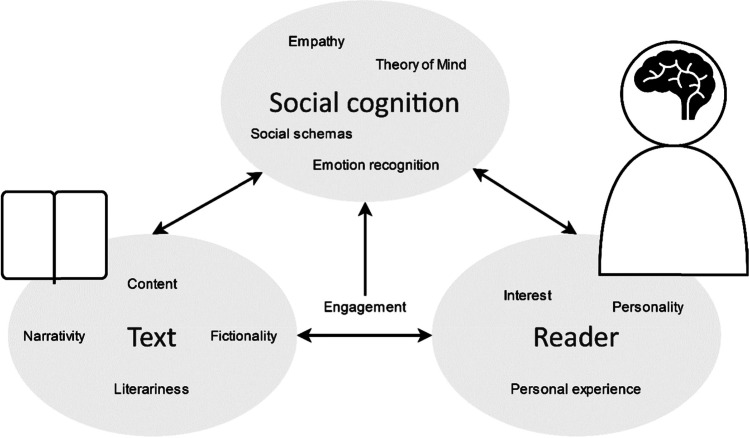
Factors of interest in the study of the social-cognitive potential of narratives and their interactions

Figure 1 graphically represents the three factors of interest in the study of the social-cognitive potential of narratives as mapped out in this article. Above, we have identified open questions related to these factors that can guide future explorations on this topic. First of all, studies should focus on unraveling the text characteristics that drive narrative effects on social cognition. A review of existing empirical work shows that most studies have focused on the general categories of fictionality and literariness (Koopman & Hakemulder, 2015), but we have argued for a shift toward studies focusing on more specific narrative textual features such as markers of perspective and characteristics of protagonists. Furthermore, future studies will benefit from integrating an individual differences approaches, as not all readers can be expected to react to a single narrative in the same way. Hence, taking into account personality characteristics such as the need for cognition or need for affect might show interesting patterns of sensitivity. Finally, deepening and broadening our view of social cognition, by developing more specific measures and investigating social-cognitive processes beyond empathy and theory of mind, will further our understanding of the specific aspects of empathy and mindreading as well as other social-cognitive abilities that narrative reading may foster.

Note that there are also relevant questions related to the interactions between these three factors that future research may study, as indicated by the arrows in Fig. 1. For example, do specific textual characteristics affect different aspects of social cognition (interaction between narrative and social cognition)? How do readers differ in the degree to which various aspects of social cognition are susceptible to improvement through narrative exposure (interaction between social cognition and reader)? Are readers sensitive to different types of narratives (interaction between narrative and reader)?

Finally, recent empirical work on the relationship between narratives and social cognition has sparked plenty of other questions and avenues for further research, such as the case of other narrative media (see Black & Barnes, 2015a, 2019; Mar et al., 2010; Nathanson et al., 2013) or even other art forms and their relationship with social cognition (for an overview, see Kou et al., 2020), the timeline of the effects of narrative exposure (see Bal & Veltkamp, 2013), and the effects of writing rather than reading narratives (e.g., Kou et al., 2020; Maslej et al., 2017). Research on these questions may also benefit from the approach outlined here, that is, by focusing on specific factors of interest, taking into account individual differences between readers (or listeners, spectators etc.), and studying a wide range of social-cognitive abilities.

To conclude, the mixed findings in the empirical literature on the relationship between narrative reading and social cognition do not warrant pessimism. Rather, they provide plenty avenues for reflection and incentives for new, carefully designed studies. Taking the research questions this review has identified as a guideline, we hope future research will unravel the circumstances that allow the social-cognitive potential of narratives to emerge.
